# Stress-induced hemorrhagic gastric ulcer after successful *Helicobacter pylori *eradication: two case reports

**DOI:** 10.1186/1752-1947-5-252

**Published:** 2011-06-29

**Authors:** Mitsuru Moriya, Akira Uehara, Toshikatsu Okumura, Mitsuaki Miyamoto, Yutaka Kohgo

**Affiliations:** 1 Department of Psychosomatic Internal Medicine, Health Sciences University of Hokkaido, Japan; 2 Uehara Clinic, Sapporo, Japan; 3 Department of General Medicine, Asahikawa Medical College, Asahikawa, Japan; 4 Department of Internal Medicine, Asahikawa Medical College, Asahikawa, Japan

## Abstract

**Introduction:**

*Helicobacter pylori *infection is a major cause of gastric ulcers, and *Helicobacter pylori *eradication drastically reduces ulcer recurrence. It has been reported, however, that severe physical stress is closely associated with gastric ulceration even in *Helicobacter pylori *-negative patients.

**Case presentation:**

We report the cases of a 47-year-old Japanese man and a 69-year-old Japanese man who developed psychological stress-induced hemorrhagic gastric ulcers, in both of whom *Helicobacter pylori *had been successfully eradicated.

**Conclusion:**

Our cases strongly suggest that not only physical but also psychological stress is still an important pathogenic factor for peptic ulceration and accordingly that physicians should pay attention to the possible presence of psychological stress in the management of patients with peptic ulcers.

## Introduction

Since Selye [1] reported that stress induces gastrointestinal ulcers, stress has been a major pathogenic factor for peptic ulceration. It is now well-documented, however, that *Helicobacter pylori *(*Hp*) infection is a major cause of peptic ulcers and that *Hp *eradication drastically reduces ulcer recurrence, thereby understating or even excluding the importance of stress in ulcer formation.

On the other hand, there is increasing evidence that peptic ulcers can occur even in patients without chronic *Hp *infection or the use of non-steroidal anti-inflammatory drugs (NSAIDs). Chen *et al*. [2] examined 32 non-*Hp *and non-NSAID duodenal ulcer cases and reported that 15.6% of the cases were closely associated with psychophysical stress. In 2009, Wong *et al*. [3] revealed that patients with *Hp*-negative idiopathic bleeding ulcers, in the pathogenesis of which mental stress might play an important role, had a high risk of mortality and recurrent bleeding.

Along these lines, we report the cases of two patients with psychological stress-induced hemorrhagic gastric ulcers in whom *Hp *had been successfully eradicated. This case report surely provides clinically important information that psychological stress should also be considered in the management of patients with gastric ulcers.

## Case presentation

### Case 1

**Figure 1:**
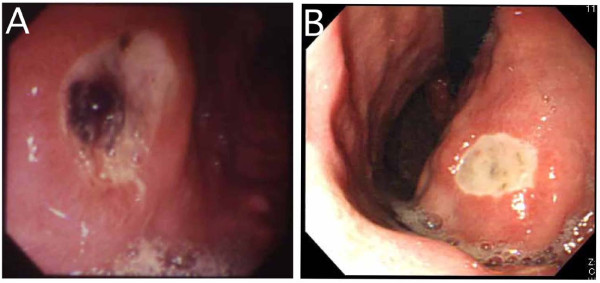
**Esophagogastroduodenoscopic findings**. **(A) **Case 1: An open gastric ulcer with an exposed vessel. **(B) **Case 2: A recurrent gastric ulcer without active bleeding.

A 47-year-old Japanese man had been admitted to the hospital for hematemesis and tarry stools 14 years ago. He had a medical history of gastric ulcer and had received *Hp *eradication therapy two years before his current presentation. Since then, he had been taking the H_2 _blocker famotidine (40 mg/day) for the prevention of ulcer recurrence. He denied use of NSAIDs or aspirin, smoking and drinking alcohol. Three days before his hospitalization he had been involved in a life-threatening accident in which his boat was overturned and he almost drowned. Afterward he was too agitated to sleep for the following three days. Laboratory findings showed mild anemia (hemoglobin 12.0 g/dL), elevated blood urea nitrogen (BUN) (27.9 mg/dL) and normal gastrin (150 pg/mL). An esophagogastroduodenoscopy demonstrated an open gastric ulcer with an exposed vessel (Figure 1A). We immediately performed endoscopic hemostasis procedures against the exposed vessel in the base of the gastric ulcer, using a heat probe coagulation method together with local injections of hypertonic saline epinephrine solution and absolute ethanol. The clinical course of the patient was uneventful after endoscopic therapy. Three *Hp-*associated examinations, that is, rapid urease test, histology and bacteriology, were all negative. As to a mind-body correlation, the patient displayed a typical type A behavior pattern [4]. The psychophysical stress he experienced in the life-threatening boat accident was so enormous that he was extremely agitated and could hardly sleep. It was speculated that this strong emotional stress induced the recurrence of hemorrhagic gastric ulcer, even though he had been under maintenance therapy with H_2 _blockade after successful *Hp *eradication.

### Case 2

A 69-year-old Japanese man visited our hospital seven years ago because of repeated tarry stools. In the previous year, he had developed a gastric ulcer and underwent *Hp *eradication therapy. He did not take NSAIDs or aspirin or drink alcohol, but he smoked an average of 20 cigarettes daily. Before he noticed tarry stools, he had been requested to edit a booklet for a community meeting. He was computer-illiterate and totally unaccustomed to such a task. He could manage to finish it by the deadline, but he developed tarry stools. He reported that his smoking pattern, including the number of cigarettes per day, was unchanged during the stressful period. Laboratory data demonstrated mild anemia (hemoglobin 11.1 g/dL), elevated BUN (33.1 mg/dL) and normal gastrin (25 pg/mL), suggesting the possible presence of gastrointestinal bleeding. Esophagogastroduodenoscopy revealed a recurrent gastric ulcer without active bleeding (Figure 1B). The absence of *Hp *infection was confirmed by negative ^13^C-urea breath test, histology and *Hp *antibody. The psychological assessment of the patient profile demonstrated that he was strict with regard to punctuality and had a strong sense of responsibility. These characteristics forced him to complete the task in which he was inexperienced by the deadline. It was therefore considered that his intense emotional stress resulted in his recurrent gastric ulcer.

## Discussion

Although it is well-established that *Hp *infection is the most important pathogenic factor in peptic ulceration, it has been reported that ulcer recurrence occurs even after *Hp *eradication. A meta-analysis of seven trials conducted in the United States showed that 20% of patients had recurrent ulcers within six months despite successful cure of *Hp *infection and no reported use of NSAIDs [5]. A similar Japanese study revealed a recurrence rate of 3% 48 months after successful *Hp *eradication [6].

The mechanism by which ulcer recurrence takes place in *Hp*-negative patients remains to be clarified, but psychological stress may be associated with its recurrence. In fact, it should be noted that the incidence of bleeding gastric ulcers significantly increased after the Hanshin-Awaji earthquake in Japan [7]. It has also been demonstrated that psychophysical stress contributed to duodenal ulcer formation among 15.7% of *Hp*-negative ulcer patients [2].

Since Selye [1] reported that stress produced the same symptoms, that is, peptic ulcer, adrenal hypertrophy and thymus atrophy, stress has been considered an important factor in the pathogenesis of ulcer formation. In fact, it has been well-documented that physical stress encountered in the intensive care unit can induce peptic ulcer bleeding among *Hp*-negative patients [8-10]. On the other hand, our two patients developed hemorrhagic gastric ulcers due to psychological stress rather than to physical pathologies. In other words, our findings strongly indicate that not only physical but also psychological stressors can cause ulcer bleeding in spite of *Hp *negativity.

## Conclusion

In conclusion, our case reports suggest that psychological stress is still an important clinical factor for peptic ulceration. In the management of patients with peptic ulcer, physicians should pay attention to the possible presence of psychological stress as well as physical causes.

## Consent

Written informed consent was obtained from the patients for publication of this case report and any accompanying images. A copy of the written consent is available for review by the Editor-in-Chief of this journal.

## Competing interests

The authors declare that they have no competing interests.

## Authors' contributions

MM, AU, TO and MM drafted the manuscript and designed the case report. All authors read and approved the final manuscript.
